# A Randomized Trial Evaluating Automated Notifications for the Identification and Treatment of Aortic Stenosis and Mitral Regurgitation: The ALERT Study

**DOI:** 10.1016/j.jscai.2025.104040

**Published:** 2025-11-18

**Authors:** Wayne B. Batchelor, Brian R. Lindman, Megan Coylewright, Antoine Keller, Miguel Sotelo, Loren Wagner, Chris Rogers, Graeme L. Hickey, Jamie Williams, Yanping Chang, Sreekanth Vemulapalli

**Affiliations:** a Inova Health System and Inova Schar Heart and Vascular, Fairfax, Virginia; b Vanderbilt University Medical Center, Nashville, Tennessee; c Cardiology Division, Essentia Health, Duluth, Minnesota; d Ochsner Lafayette General, Ochsner Health, Lafayette, Louisiana; e Tempus AI, Chicago, Illinois; f Medtronic, Minneapolis, Minnesota; g Division of Cardiology, Duke University Department of Medicine, Duke Health, Durham, North Carolina

**Keywords:** aortic stenosis, automated notifications, clinician decision support, electronic health record, health equity, mitral regurgitation

## Abstract

**Background:**

Aortic stenosis (AS) and mitral regurgitation (MR) are common cardiac conditions associated with significant morbidity and mortality if left untreated. Despite the availability of effective therapies, many patients with severe AS and MR do not receive timely interventions, with disparities particularly affecting minority populations, women, and those in rural areas.

**Methods:**

The Addressing undertreatment and heaLth Equity in aortic stenosis and mitral regurgitation using an integrated ehR plaTform (ALERT) study is a multicenter, prospective, cluster-randomized controlled trial designed to evaluate the impact of automated electronic health record notifications on the management of severe AS and MR. This cluster-randomized controlled trial involves at least 5 US hospital systems. Providers are randomized to receive automated notifications or not receive notifications, based on echocardiogram report findings. The primary end point is a hierarchical composite of transcatheter or surgical valve intervention (valve intervention) or multidisciplinary heart team clinic visit within 90 days. The study aims to randomize at least 600 providers and 1500 total patients 1:1 into each study arm, hypothesizing that automated notifications increase the proportion of patients receiving appropriate evaluation and treatment.

**Results:**

The results of the study are pending completion of enrollment and should be available in Q2 2026.

**Conclusions:**

The ALERT study leverages integrated electronic health record platforms to address undertreatment in severe AS and MR, with the potential to improve patient outcomes and reduce disparities in care.

## Introduction

Aortic stenosis (AS) and mitral regurgitation (MR) are prevalent cardiac valvular conditions that pose significant health risks if left untreated.^1^ The AS is highly prevalent among older adults, and estimates suggest that nearly half of the patients with symptomatic severe AS do not receive aortic valve replacement (AVR).^2^ The average life expectancy of those left untreated after the onset of symptoms is approximately 2 years, underscoring the critical need for timely intervention.^3^ Similarly, MR, which affects 2% to 10% of the general population and increases with age,^3^ is often underdiagnosed and undertreated. It is estimated that up to 50% of patients with symptomatic severe MR do not receive surgical or transcatheter intervention,^4^ resulting in a poor prognosis with a median life expectancy of only 5 years following symptom onset.^5^

Treatment gaps for AS and MR are even more pronounced in racial and ethnic minority populations. Although minorities represent approximately 20% of the US population over the age of 75 years,^6^^,^^7^ only 9% of transcatheter aortic valve replacement (TAVR) patients self-identify as being a minority.^8^ Factors, such as lower disease prevalence reported in minority populations, may contribute to differences in AVR utilization, but do not fully explain the treatment gap.^9^, ^10^, ^11^ Additionally, underutilization of TAVR has been reported among women^12^ and patients living in areas with lower population density.^13^ Multiple reasons for the lower rate of referral and treatment have been cited, including provider specialty, differences in disease prevalence, patient characteristics, health care access, bias, and health care system factors.^11^^,^^14^^,^^15^ Therefore, there exists a need to better identify patients with AS and MR who are potential candidates for surgical and transcatheter therapies to ensure they are evaluated and, when warranted, treated in a timely manner.

The Addressing undertreatment and heaLth Equity in aortic stenosis and mitral regurgitation using an integrated ehR plaTform (ALERT) study^16^ aims to evaluate how electronic health record (EHR) platforms can be leveraged to address the undertreatment of patients with severe AS and moderate-to-severe or severe MR. The ALERT study will enroll patients from at least 5 US hospital systems with racially and ethnically diverse populations. Patients will be included in the study if they have either severe AS or moderate-to-severe or severe MR and are considered not managed for their disease at the time of their echocardiogram. The end point will be a hierarchical composite of transcatheter or surgical valve intervention or multidisciplinary heart team (MHT) clinic visits within 90 days from the date the notification goes out or would go out.

## Materials and methods

### Study design

**Figure 1:**
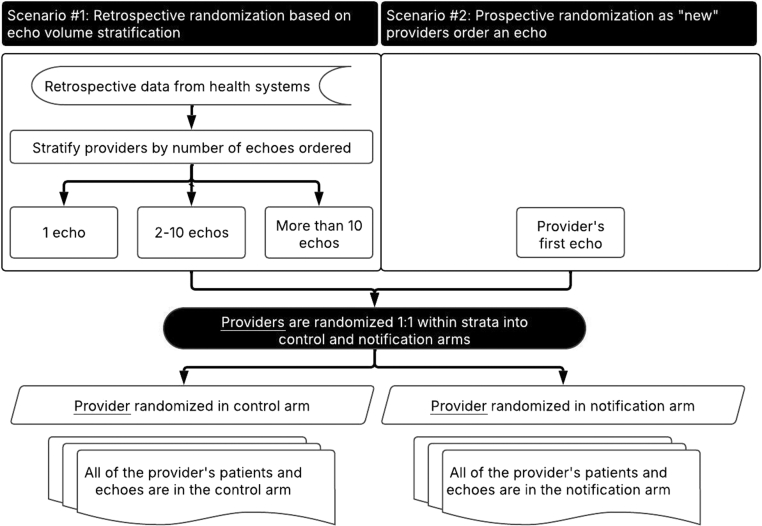
**Randomization process.** Providers will be randomized in 2 tranches. The first tranche will be providers for whom retrospective data on their historical echo volumes are available. These providers will be stratified by their historical echo volume. Randomization will be 1:1 into control and notification arms within the echo volume strata. The second tranche will be providers randomized 1:1 upon ordering an echo for an eligible patient. Providers stay in their respective study arm for the duration of the study. Only patients who meet the eligibility criteria are included in the study, and providers only receive notifications on those patients.

**Figure 2:**
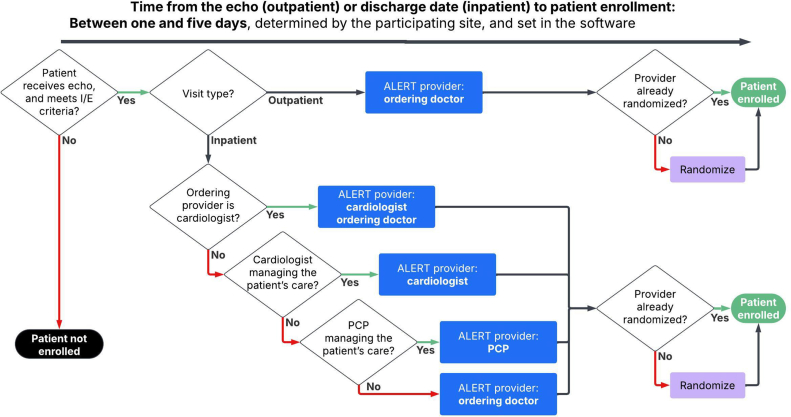
**Upon the date of the echocardiogram or the discharge date, for outpatients and inpatients, respectively, decision criteria are run to determine the provider assigned to the patient for the study.** For outpatients, it is the ordering provider; for inpatients, a hierarchy is considered to prioritize a cardiologist or a primary care provider (PCP). Once the provider is assigned to the patient, they are randomized into study arms if they have not yet been randomized. The randomization of the provider dictates the randomization of the patient. I/E, inclusion/exclusion.

The ALERT study is a multicenter, prospective, cluster-randomized controlled trial designed to evaluate the impact of automated notifications on the identification and management of patients with severe AS or moderate-to-severe or severe MR. This study involves at least 5 US hospital systems and will focus on patients who are potentially suboptimally managed insofar as they have not undergone procedural treatment or been referred to an MHT. Providers, including doctors and advanced practice providers, will be randomized to either receive automated notifications or not receive notifications for the duration of the study, ensuring a reduction in cross-contamination and enhancing the precision of the alert intervention (Figure 1). Notifications will be delivered based on an algorithm to the most appropriate provider, aiming to spur timely follow-up evaluations and interventions (Figure 2). The study’s primary end point will be a hierarchical composite of time to transcatheter or surgical valve intervention or MHT clinic visits from the date the notification goes out or would go out. The rationale for the 90-day observation window is that a shorter time frame facilitates easier inference of causality regarding the effectiveness of the notification, and it aligns with recent performance and quality measures outlined for AS and MR.^17^ Additionally, the hierarchical composite end point ensures that even if some patients are not immediately indicated for a valve intervention, prompting a referral for MHT evaluation is consistent with professional guidelines.^18^

The study duration spans from the date of the first patient enrollment to the date of last follow-up and study completion. The patients’ monitoring window starts between 1 and 5 days after the echocardiogram (for outpatients) or after the discharge date (for inpatients). This is henceforth referred to as the index date. The specific day the notification is sent is when the monitoring window begins and is determined by the participating site and set in the software.

The study will be conducted according to good clinical practice principles, and it conforms to the Declaration of Helsinki. The study protocol was reviewed and approved by an independent institutional review board (IRB) before study initiation and will continue to be reviewed by the IRB for the duration of the study (Advarra IRB Pro00071710). A waiver of consent for the patient and provider will be obtained by the central IRB. Each site will be given the autonomy to bring awareness of the study to providers who might receive a notification. This autonomy was purposefully allowed to aid in implementation and take into account differing practice patterns with regard to referral and institutional governance, with the understanding that each site will adhere to the study protocol. The majority of the sites conducted an “awareness communication campaign” that included an IRB-approved informational letter with opt-out instructions. However, one site elected to forgo any prestudy communication at all and started alerting without warning. This approach was approved by their institution's IRB. Each site implemented this process by its own design, and it was the site principal investigators’ responsibility to align with their IRB and strategy for their institution. All study sites follow and comply with the principles of the Declaration of Helsinki, 21 CFR Part 11 (electronic records, electronic signatures), the clinical trial agreements, the procedures described within this clinical investigation plan, and local ethical committee requirements. The study will be conducted according to federal, national, and local laws, regulations, standards, and requirements of the countries where the study was conducted. The ALERT study is registered at ClinicalTrials.gov (NCT06099665).

### Study and site oversight

This study utilizes a steering committee that advises on the scientific content of the study and provides input for study execution, aiming to provide unbiased opinions and expertise to the clinical study design and process. Each participating site designates a site principal investigator to oversee the execution of the study at their site. Medtronic’s research divisiempus and the steering committee with expert guidance, protocol review and validation, study design, peer review of the analysis programming, and final analysis validation. It should be noted that Medtronic is providing funding for this study in a collaborative research arrangement with Tempus AI. For clarity, Tempus AI is the sponsor of the study. Medtronic’s involvement also includes providing scientific input and oversight through its research division, including protocol review, analysis validation, and participation in the steering committee.

### Study objective and hypothesis

The study aims to test whether the use of automated notifications will affect the proportion of patients who are appropriately evaluated and, when warranted, treated with a valve intervention.

Our hypothesis is that patients with severe AS or moderate-to-severe or severe MR who are in the care of physicians receiving the automated notifications will be more likely to undergo a valve intervention or have a clinic visit with the MHT within 90 days compared to those whose physicians do not receive the notifications (Central Illustration).

**Central Illustration:**
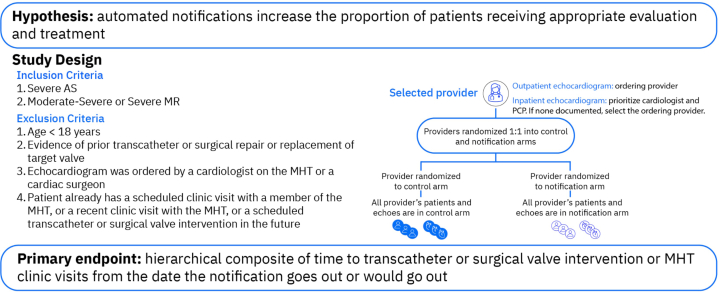
**A randomized trial evaluating the impact of automated notifications on the identification and treatment of patients with aortic stenosis (AS) and mitral regurgitation (MR): Design of the ALERT study.** MHT, multidisciplinary heart team; PCP, primary care provider.

### Study population

Patients who meet 1 or more criteria for severe AS or moderate-to-severe or severe MR (Table 1), but do not have an associated follow-up visit scheduled with a cardiologist member of the MHT, cardiovascular surgeon, or have a valve intervention scheduled, will be included in the study. Patients with both AS and MR will have an alert sent for AS first. Patients must be at least 18 years old and have an echocardiogram ordered by a provider who is not part of the MHT nor a cardiac surgeon. Patients with a nonnative aortic or mitral valve (bioprosthetic or mechanical) will be excluded if that nonnative valve was identified with criteria for disease, because this study is specifically designed to evaluate care pathways for patients with native valve disease, given that concerns about undertreatment have primarily focused on this population. However, if a patient is identified with AS but has a mitral valve prosthesis, they will still be considered for the study. The opposite scenario, with a patient identified with MR but having an existing AS prosthesis, is true as well. The exclusion occurs if the target valve identified with disease has been previously treated with a therapeutic device or procedure. In addition, patients already scheduled for a recent valve intervention or a clinic visit with the MHT are excluded from the study (Table 2). This includes recent visits in the past, after the study start date, or future visits or procedures already scheduled for the patient.

**Table 1:** Inclusion criteria.

At least 1 of the following 3 options for aortic stenosis and/or the single option for MR is determined from echocardiogram findings: 1. AVA or DI (patients with either AVA or DI measured in their echo, as well as at least 1 hemodynamic measure above the minimum threshold) a. Either: i. AVA ≤1.0 cm^2^ ii. DI ≤0.25 b. And any of i. Aortic mean gradient ≥15 mm Hg ii. Aortic peak gradient ≥30 mm Hg iii. Aortic jet velocity ≥2.75 m/s 2. AVA + other (patients with AVA and at least 1 other echo measurement in their echo above the threshold) a. 1.0 cm^2^ < AVA ≤ 1.2 cm^2^ b. And any of: i. Aortic mean gradient ≥40 mm Hg ii. Aortic peak gradient ≥64 mm Hg iii. Aortic jet velocity ≥4.0 m/s 3. POSSIBLE (possible aortic stenosis, but requires human review) a. AVA is null or >1.2 cm^2^ b. And any of: i. Aortic mean gradient ≥ 40 mm Hg ii. Aortic peak gradient ≥ 64 mm Hg iii. Aortic jet velocity ≥4.0 m/s Or a. Either i. AVA ≤1.0 cm^2^ ii. DI ≤0.25 b. And all are: i. Aortic mean gradient <15 mm Hg ii. Aortic peak gradient <30 mm Hg iii. Aortic peak velocity <2.75 m/s Any patient flagged for "POSSIBLE" does not result in automatically notifying the provider; instead, a notification is sent to the Tempus research team for manual review in conjunction with the site principal investigator, and if requested by Tempus or the site principal investigator, a steering committee member. If clinical confirmation is received for severe aortic stenosis from the site principal investigator, a notification is sent to the provider. 1. MR a. Mention of severe MR or moderate-to-severe MR (based on a qualitative characterization of MR severity and not based on quantitative measurements) Any patient mentioned with only moderate MR or a lesser severity will be excluded.

AVA, aortic valve area; DI, dimensionless index; MR, mitral regurgitation.

**Table 2:** Exclusion criteria.

Exclusion criteria: 1. Age <18 y. 2. The patient had evidence of a prior transcatheter or surgical repair or replacement of the target valve. 3. The echocardiogram was ordered by a cardiologist on the MHT or a cardiac surgeon. 4. Patient already has a scheduled clinic visit with a member of the MHT or a recent (since the start of the study at that site) clinic visit with the MHT, or a scheduled transcatheter or surgical valve intervention in the future.

MHT, multidisciplinary heart team.

### Sample size

Based on retrospective data within the Tempus Next database (Tempus AI, Inc), a cloud-hosted, HIPAA-compliant, artificial intelligence–enabled care pathway platform that notifies providers of deviations from guideline-based care, it is estimated that 35% of patients with severe AS or moderate-to-severe or severe MR will meet the criteria for the study. The exclusion of managed and treated patients, and the 90-day observation window, resulted in an expected incidence of the end points to be 5% and 10% for valve intervention and MHT, respectively. Given these assumptions, the study has 82% power to detect a 5% absolute increase in the proportion of patients who receive MHT follow-up or valve intervention within 90 days with a sample size of 1246 patients and 500 providers/clusters.

The power of the study was determined through simulations using exponential distributions for the time to valve intervention and referral to MHT within a Gumbel-Hougaard copula. The simulations assumed an absolute effect of the notifications on the rate of valve intervention or referral to MHT, increasing their incidence within 90 days by 5%. The study aims to randomize at least 600 providers, caring for 1500 total patients, 1:1 into each study arm across 5 sites in the US.

### Randomization

Randomization occurs at the level of the provider ordering the echocardiogram. Providers ordering echocardiograms with results meeting the inclusion/exclusion criteria (Tables 1 and 2) will be randomized on a 1:1 basis to either receive automated notifications or not receive notifications for all their patients with echocardiograms meeting the inclusion criteria. Individual patients are not randomized. Once a provider is randomized into either the notification or no notification arm, that assignment will remain unchanged for the duration of the study (Figure 1).

To minimize contamination between study arms, providers were randomized and remained in their assigned arm for the duration of the study, and sites were encouraged to limit communication about the intervention. Notification delivery and provider actions will be monitored to detect potential cross-over.

To minimize confounding due to variability in practice patterns and resources, randomization was stratified by provider historical echo volume within each site for the first tranche of providers, and by site for all providers. This approach was intended to balance case mix and ensure comparability between study arms.

### Notification

**Figure 3:**
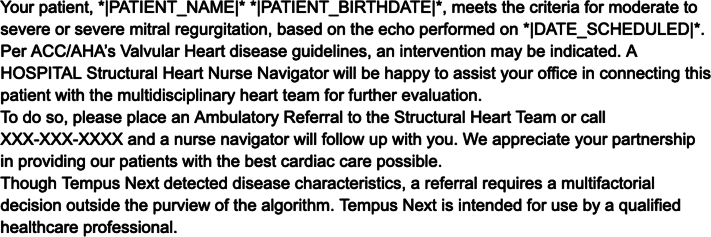
**Example of a notification to the provider in the notification arm.** ACC, American College of Cardiology; AHA, American Heart Association.

In the notification arm, providers will receive an automated notification generated by Tempus Next software when a patient is identified by the software via review of data entered into the hospital’s EHR system who meets all inclusion criteria and exclusion criteria. The notification includes the patient’s name, medical record number, date of the echocardiogram, and a summary of the echocardiographic findings consistent with severe AS or moderate-to-severe or severe MR. The notification also includes guideline-based recommendations for further evaluation and management (Figure 3).

For providers randomized into the notification arm, notifications will be delivered between 1 and 5 days after the index date. The specific day the notification is sent is determined by the participating site and set in the software. The recipient logic is shown in Figure 2. For outpatients, the assigned provider in the ALERT study is the provider who ordered the echocardiogram. If the provider is randomized to the notification arm, they will receive the notification. For inpatients, the determination of the assigned provider will follow a hierarchy as follows:

1. The cardiologist who ordered the echocardiogram. If a cardiologist did not order the echo, then proceed to the next option.
2. The cardiologist who is managing the patient’s care will be determined by previous provider documentation from the EHR. If no cardiologist is identified, proceed to the next option.
3. The primary care provider will be determined by previous provider documentation from the EHR. If there is no primary care provider determined to be affiliated with the patient’s care, then proceed to the next option.
4. The provider who ordered the echocardiogram.

We acknowledge that the primary care provider field in the EHR may not always accurately reflect the provider actively managing the patient’s care, particularly in the inpatient setting. Although our protocol prioritized the primary care provider after the managing cardiologist, future implementations of this approach may benefit from prioritizing the provider who ordered the echocardiogram over the primary care provider to improve accuracy in notification delivery.

Notifications for inpatient echocardiograms will be sent only if the patient did not receive an aortic or mitral valve intervention during the same hospital stay.

The 90-day observation window will begin on the day of the patient’s index date. Each site will have the opportunity to customize how the notification will be formatted within the EHR, so long as the notification includes the required language and timing as set forth in the research protocol. Any additional customizations will require approval by the research steering committee. Customizations also include modifications to the institution's EHR to enable a more efficient response to the notification by the provider, such as a one-click order feature for ordering a referral to the MHT.

### Study outcomes

The primary end point will be a hierarchical composite of the following: (1) time to transcatheter or surgical valve intervention for severe AS or moderate-to-severe or severe MR (performed within the first 90 days from the index date), or (2) time to a clinic visit with at least 1 member of the MHT within the first 90 days from the index date.

Secondary outcomes will include each of the individual components of the hierarchical primary end point: procedural treatment and clinic visit with the MHT.

### Subgroup analysis

We will evaluate how socioeconomic demographic characteristics of patients with severe AS or moderate-to-severe or severe MR affect rates of treatment and times to clinic visit within 90 days (age, sex, race, ethnicity, home ZIP Code, payer). The subgroup analyses will consider stratifications by race, sex, and site groups.

### Statistical considerations

Following Dong et al,^19^ the primary end point will be analyzed using a stratified win ratio statistic, a hierarchical composite of the times to valve intervention and MHT referral. The win ratio estimator is a method used to compare outcomes between 2 groups in a study, specifically the notification arm and the control arm. This procedure is as follows:

1. Each patient in the notification arm is compared to every patient in the control arm.
2. For each comparison, the outcome of the patient in the notification arm is considered better if:
  a. The patient in the notification arm undergoes valve intervention before the patient in the control arm does, or before the control patient is censored (ie, no longer observed in the study).
  b. If neither patient undergoes valve intervention before censoring, the patient in the notification arm has a visit for monitoring heart health (MHT visit) before the control patient does, or before the control patient is censored.

These comparisons will be categorized into 5 groups: (1) the notified patient had valve intervention first, (2) the control patient had valve intervention first, (3) the notified patient had an MHT visit first, (4) the control patient had an MHT visit first, and (5) none of the above conditions are met. If a comparison falls into the first or third group, the outcome for the notified patient is considered better, and this counts as a win for the notification arm. The total number of wins for the notification arm and the control arm is then calculated separately. The win ratio is determined by dividing the total number of wins for the notification arm by the total number of wins for the control arm. This ratio provides a measure of how often the notification arm’s outcomes are preferred over the control arm’s outcomes.

### Tempus Next platform

**Figure 4:**
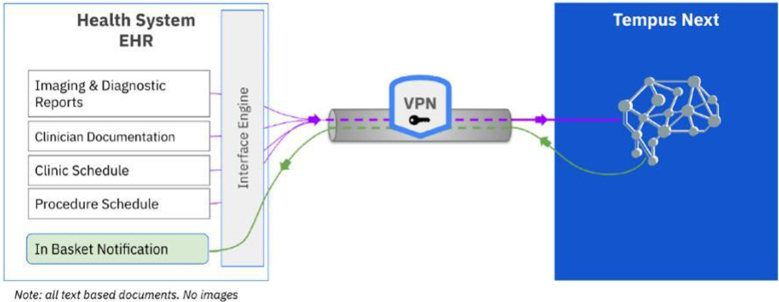
**Textual reports, diagnostic information, clinic notes, and schedules flow from the health system’s electronic health record (EHR) to the Tempus Next platform.** Screening for patients takes place in this standardized database to harmonize data across other health systems and EHR.

Health level 7 interfaces were created to receive digital imaging and communication, picture archiving and communication systems, procedure schedules and clinic visits, and EHR data using the Tempus Next platform. The software is designed to augment cardiac care management and reduce the undertreatment of cardiovascular disease. It functions by automating the detection of patients with cardiovascular disease and helps to optimize care pathways for these patients through a web-based user portal and as a clinical decision support tool through EHR notifications. Disease detection is accomplished through the data extraction from echocardiogram reports. These reports contain both structured and unstructured data, and the algorithm utilizes NLP to extract the target measurements of the aortic valve for AS and the description of moderate-severe or severe MR from the “findings section” of the report. The software has demonstrated high accuracy in detecting moderate or severe AS, with a recent study at 2 US hospital systems showing a detection accuracy of 98.6% and detection precision of 92.9%.^20^ A schematic of the data flow is shown in Figure 4.

### Data collection and monitoring

Data from the hospitals’ EHR will be continuously sent to Tempus Next via a health level 7 feed or using other secure methods approved by the site. Data points to be collected include raw echocardiogram reports, which will provide key data elements for aortic valve area (AVA), dimensionless index, mean gradient, peak gradient, peak velocity, and left ventricular ejection fraction. The following data points are contributed as discrete data elements from the health level 7 interfaces or calculated by Tempus Next using discrete data elements: New York Heart Association class, age, sex, race, ethnicity, home ZIP Code, specialty of the ordering/referring provider, provider’s randomization arm, comorbidities, payer, whether a notification was sent, date and time the notification was sent, mortality, heart failure hospitalization, follow-up echocardiogram, time to follow-up echocardiogram, and time to MHT visit. The accuracy of data extraction and completeness is verified during the preenrollment quality assurance check with each site’s principal investigator. Random patient review of at least 10 patients is conducted to ensure data are accurate and being analyzed by Tempus Next correctly before starting enrollment and sending alerts.

There will be a 90-day observation period after the notification date to evaluate the impact of notifications on treatment with a heart valve procedure or clinic visits with the MHT. If a patient has more than 1 echo that meets the criteria, each echo will be handled individually and will trigger (or not trigger) a notification based on whether the provider assigned to that echo is randomized in the control or notification arm. If that patient’s repeat echo occurs within 90 days, the subsequent echo will censor the first echo’s observation period.

## Discussion

If left untreated, severe AS and MR lead to increased morbidity and mortality. Even though effective and guideline-recommended transcatheter and surgical treatments are available, a substantial proportion of patients with severe symptomatic AS and MR do not receive timely intervention.^15^^,^^21^ Racial and ethnic minority groups, women, and patients living in areas with lower population density are particularly vulnerable to underdetection and undertreatment.^22^, ^23^, ^24^, ^25^, ^26^

The ALERT study aims to evaluate how automated notifications support the identification and evaluation of patients potentially indicated for a transcatheter or surgical valve intervention to treat severe AS or moderate-to-severe or severe MR. The primary impetus is to address the significant gaps in the recognition and management of severe AS and MR, which lead to poor patient outcomes and increased health care disparities. By leveraging advanced artificial intelligence and integrated EHR platforms, we hope to improve the timely and appropriate treatment of high-risk patients, ultimately enhancing patient outcomes and reducing health care disparities.

Although it is anticipated that automated alerts will increase the rate of the composite end point, the degree of improvement, the impact on different patient subgroups, and the operational challenges of implementation (such as alert fatigue and provider engagement) remain uncertain. The ALERT study is designed to rigorously quantify these effects in a real-world, multicenter, randomized setting, providing generalizable evidence to inform future quality improvement initiatives.

We acknowledge that the clinical indications for intervention differ between severe AS and moderate-to-severe or severe MR, and that further evaluation is often required for MR patients. The inclusion of both conditions reflects the need to address undertreatment across the spectrum of valvular disease, but we will perform subgroup analyses to assess whether the effect of notifications differs by valve type. The primary end point’s inclusion of an MHT clinic visit ensures alignment with guideline-based care, which emphasizes multidisciplinary evaluation for both AS and MR.

A single-center recent cluster-randomized controlled trial reported significant increases in the rates of AVR following notifications to the providers of patients with AS, when compared with patients in the usual care arm.^27^ The notification effect was highest among women, patients aged over 80 years, and patients with a transthoracic echocardiogram performed in the inpatient setting. An important distinction in our study is that the former study enrolled all adult patients with an AVA ≤1 cm^2^, whereas the current study requires a high gradient in conjunction with the low AVA. This was intended to minimize the impact erroneous AVA measurements may have on the grading of AS.^28^ Additionally, care must be taken to avoid “notification fatigue” by limiting notifications only to providers of patients with the disease who would not have otherwise received treatment or referral to a valve specialty.^29^ This study excludes patients already managed for their disease. Lastly, although notifications are intended to spur a follow-up, a subsequent referral to the MHT, and an intervention, the impact of the notification, even in the setting of a randomized clinical trial, could be diluted as time goes on. Our study’s observation period is within 90 days of the index date (the date of the notification or when the notification would have been sent), with the intention of increasing our sensitivity in detecting the impact of the notification on the end point immediately after the notification is sent. The inclusion of undertreated and unmanaged patients and the shorter observation window result in the expected incidence of the end points to be 5% and 10% for valve intervention and MHT, respectively.

The ALERT study employs a multicenter, prospective, cluster-randomized controlled trial design to evaluate the impact of automated notifications on the management of severe AS and MR. This approach is unique in several ways. The experimental design of the study removes bias and helps to ensure causal inference. Operationally, automated notifications are generated based on echocardiogram findings, providing providers with timely and actionable information to support the identification and evaluation of patients potentially indicated for a valve intervention. Additionally, the software integrates seamlessly with hospital EHR systems, allowing for continuous data collection and real-time notifications without disrupting clinical workflows. The agnostic nature of the software may help prevent the diagnostic and therapeutic biases that have historically disadvantaged underrepresented groups in the management of valvular heart disease.^11^ Performing randomization at the level of the provider ordering the echocardiogram helps reduce the risk of cross-contamination when compared to patient-level randomization and ensures that providers consistently receive or do not receive notifications for all their patients meeting the study criteria. Lastly, the study includes prespecified subgroup analyses to evaluate disparities in care across different races, ethnicities, sexes, and site groups, and factors associated with disparities in care. This focus on health equity is critical to addressing the broader issues of undertreatment and health care disparities.

### Limitations

Our study has several limitations. Despite stratified randomization, residual confounding due to differences in practice patterns, referral networks, and resources between providers or sites may persist. There is variability in the implementation of the study across participating sites, including differences in provider awareness of the study (informed consent), the wording of the notification, and the presence or absence of tools designed to facilitate referral to the MHT (eg, referral buttons). Although such heterogeneity may introduce confounding and limit internal validity, it also enhances the external validity of the findings by reflecting the real-world conditions under which the intervention would be deployed. Additionally, the effectiveness of the automated notifications depends on the engagement and responsiveness of the providers receiving them.

There is a risk that some notifications may be overlooked or not acted upon due to “notification fatigue” or other factors, which could bias results toward the null. To mitigate notification fatigue, notifications were limited to patients not already managed or scheduled for follow-up, and each notification included clear, guideline-based recommendations. The notification format and delivery were customized in collaboration with each site to ensure seamless integration into provider workflows and minimize disruption.

Although an automated referral to the valve clinic could further reduce provider-level bias, our study was designed to evaluate the impact of provider-directed notifications, which is more readily implementable across diverse health systems. This approach allows for provider judgment and respects institutional workflows, but future studies may be warranted to assess the incremental benefit of direct automated referrals.

The study population reflects the patients evaluated within the participating hospital systems, which may limit the generalizability of the findings to other settings or populations. Additionally, the operational challenges of implementing EHR-based randomization and data access for research purposes are substantial and may limit scalability in some environments. To improve generalizability, we are enrolling in 5 to 6 hospital systems across a demographically diverse population in the US.

The study relies on EHR data for follow-up and outcome assessment, which may not capture all relevant clinical events or patient interactions, such as evaluations or interventions performed at outside institutions, potentially underestimating outcomes. Future studies could address this limitation by linking to regional or national claims registries.

Lastly, the 90-day observation period may not be sufficient to fully assess the long-term impact of the notifications on patient outcomes. The 90-day follow-up period was selected to facilitate causal inference and align with quality measures, but may miss delayed interventions or evaluations. Future analyses with extended follow-up (eg, 6 to 12 months) would provide additional insight into the longer-term impact of the intervention.

## Conclusion

The ALERT study is a cluster-randomized trial designed to evaluate the effectiveness of automated notifications to improve the treatment and referral of patients with clinically significant aortic and mitral valvular heart disease within a real-world clinical environment. The proposed intervention to improve processes and systems of care is highly scalable, minimally reliant on human time/effort to operationalize, and could reduce bias in the system that yields disparities in care. Collectively, such an intervention could improve quality for all patients with valvular heart disease, which would be predicted to optimize patient outcomes and reduce costs to the system related to delayed and neglected treatment.
